# Saccade Latency Provides Evidence for Reduced Face Inversion Effects With Higher Autism Traits

**DOI:** 10.3389/fnhum.2019.00470

**Published:** 2020-01-24

**Authors:** Robin Laycock, Kylie Wood, Andrea Wright, Sheila G. Crewther, Melvyn A. Goodale

**Affiliations:** ^1^School of Health and Biomedical Sciences, RMIT University, Melbourne, VIC, Australia; ^2^School of Psychology and Public Health, La Trobe University, Melbourne, VIC, Australia; ^3^The Brain and Mind Institute, The University of Western Ontario, London, ON, Canada

**Keywords:** autism, face processing, face inversion, saccade, eye-movements

## Abstract

Individuals on the autism spectrum are reported to show impairments in the processing of social information, including aspects of eye-movements towards faces. Abnormalities in basic-level visual processing are also reported. In the current study, we sought to determine if the latency of saccades made towards social targets (faces) in a natural scene as opposed to inanimate targets (cars) would be related to sub-clinical autism traits (ATs) in individuals drawn from a neurotypical population. The effect of stimulus inversion was also examined given that difficulties with processing inverted faces are thought to be a function of face expertise. No group differences in saccadic latency were established for face or car targets, regardless of image orientation. However, as expected, we found that individuals with higher autism-like traits did not demonstrate a saccadic face inversion effect, but those with lower autism-like traits did. Neither group showed a car inversion effect. Thus, these results suggest that neurotypical individuals with high autism-like traits also show anomalies in detecting and orienting to faces. In particular, the reduced saccadic face inversion effect established in these participants with high ATs suggests that speed of visual processing and orienting towards faces may be associated with the social difficulties found across the broader autism spectrum.

## Introduction

A core element of Autism Spectrum Disorder (ASD) is a difficulty in dealing with social situations, including deficits in eye-contact and reading non-verbal social signals. In addition, individuals with ASD often show impairments in attending to social information, spending less time, for example, fixating on the eyes and the central region of faces (Snow et al., 2011). Impairments in identifying emotions from facial expressions (Lozier et al., 2014), and based on eye-tracking studies, in shifting attention towards Guillon et al. (2016), and disengaging attention away from faces (Chawarska et al., 2010; Kikuchi et al., 2011) have also been reported in ASD populations.

Given that nonverbal social cues are usually rapid and dynamic, speed of visual processing and the orienting of attention may be important for developing social-communication skills. For example, the latency of the event-related potential (ERP) associated with early perceptual face processing has been shown to predict emotion recognition in adolescents with ASD (Lerner et al., 2013). In addition, under free-viewing of photographs of natural scenes, individuals with ASD are slower to first fixate on a person in the scene, and spend less time looking at a person when there are other objects in the scene (Wilson et al., 2010).

In neurotypical populations, faces appear to constitute a unique category of objects that gain priority access to neural processing possibly *via* a direct superior colliculus-pulvinar-amygdala route (McFadyen et al., 2017). For example, faces capture attention in visual search, even when they are irrelevant to the task (Devue and Grimshaw, 2017), and infants also demonstrate preferential looking at faces (Johnson et al., 1991). The salient nature of faces is well illustrated by a saccadic choice reaction time paradigm employed by Crouzet et al. (2010) in which saccade onset times towards photographs of natural scenes presented simultaneously to the left and right of fixation averaged 150 ms when detecting the side containing a face (the express saccade range), compared to more than 180 ms when they were detecting a vehicle. This speed of visual processing for faces is consistent with reports of rapid ventral stream activation (Braeutigam et al., 2001; Eimer and Holmes, 2002; Liu et al., 2002), or may reflect direct subcortical pulvinar pathways to the amygdala that bypass early visual cortex (Johnson, 2005; Méndez-Bértolo et al., 2016; McFadyen et al., 2017). Interestingly, this type of fast processing is likely mediated by the magnocellular system which is also reported to be impaired in ASD (Greenaway et al., 2013). In the present study, we used the Crouzet et al.’s (2010) paradigm to examine the detection of faces vs. cars to disentangle whether the deficit in face detection in ASD is specific to social stimuli such as faces or instead reflects a general deficit in saccadic orienting.

The ASD literature on visually guided saccade tasks has been somewhat inconsistent with regard to latency measures. These studies involve the onset of a single simple target such as a small square [or a more engaging target such as a smiley face or a clown for children participants (Kelly et al., 2013; Kovarski et al., 2019)], triggering a reflexive visually guided saccade, and have found ASD to be associated with slower saccadic onset times (SOTs) towards a target (Goldberg et al., 2002; Miller et al., 2014; Wilkes et al., 2015), whilst others appear to suggest intact (Minshew et al., 1999; Takarae et al., 2004; Luna et al., 2007; Kelly et al., 2013; Zalla et al., 2018) or even faster (Kovarski et al., 2019) saccadic latency in ASD. In the current study however, where two competing photographs were presented, we were interested in the speed of saccadic orienting towards the image that contained the target category. Given that faces are less salient in those with ASD (Chawarska et al., 2010; Kikuchi et al., 2011; Guillon et al., 2016) it would be expected that utilization of a choice reaction time task should be associated with slower saccadic responses to face targets in ASD populations.

In addition, we also examined the effect of face inversion. In typical observers, recognition of inverted faces is reported to be more difficult, possibly due to the disruption of holistic face processing that occurs with inversion (Rossion, 2009). Reduced face inversion effects have been reported in the ASD literature (Rose et al., 2007) and have also been found to predict autism traits (AT) in a neurotypical population (Wyer et al., 2012). A 2012 systematic review argued there is insufficient evidence for reduced inversion effects in ASD (Weigelt et al., 2012). However, while most face inversion studies in those on the autism spectrum have measured identity recognition, only a few have utilized electrophysiological measurements to assess more automatic aspects of face processing, finding reduced inversion effects in ASD (McPartland et al., 2004; Vettori et al., 2019). Here, we measured saccadic latency when participants were detecting upright and inverted faces or cars which may also provide a more reflexive measure of attentional bias towards faces.

As a first step to testing whether or not “reflexive” saccadic mechanisms mediating the rapid detection of faces are compromised in ASD, we adopted the dimensional approach to investigating the autism spectrum (Landry and Chouinard, 2016). Similar to patterns observed in ASD samples, the broader autism phenotype, which includes family members of those with an ASD as well as neurotypical samples with higher ATs, has demonstrated anomalies in a number of cognitive processes, including executive function (Christ et al., 2010), visual processing (Crewther et al., 2015; Cribb et al., 2016), face emotion processing (Palermo et al., 2006; Wallace et al., 2010; Spencer et al., 2011) and in eye-movement patterns towards faces (Davis et al., 2001; Åsberg Johnels et al., 2017). Together, these results suggest that this dimensional approach provides a useful model of ASD, and may shed light on the similarities and dissimilarities between clinical and sub-clinical populations. In the current study, therefore, we tested individuals with high autism-like personality traits drawn from the general population. Such individuals were identified using a self-report scale that treats ASD as one end of a spectrum of behavioral traits extending into the general population (Kanne et al., 2012).

We predicted that individuals with high autism-like traits would exhibit the slower onset of saccadic eye movements than individuals with lower autism-like traits when detecting faces, even though the performance of the two groups might not differ when detecting inanimate objects, such as cars. We also predicted that individuals with high autism-like traits would demonstrate reduced saccadic face inversion effects compared to individuals with low ATs, but neither group would show a “car inversion” effect.

## Materials and Methods

### Participants

Forty-seven participants (32 females, 15 males; mean age = 25.7, *SD* = 6.3) with normal or corrected to normal vision, were tested after providing written informed consent. All procedures were approved by the La Trobe University Human Ethics Committee and carried out in accordance with the approved protocol and relevant regulations. Prior to completing the experiment, participants completed an online version of the Subthreshold Autism Trait Questionnaire (SATQ; Kanne et al., 2012), a 24 item self-report questionnaire assessing a broad range of ATs encompassing social-communication and restricted or repetitive behaviors that has good test-retest reliability, internal consistency and discriminant validity (Nishiyama and Kanne, 2014). A factor analysis revealed five factors (social interaction and enjoyment, oddness, reading facial expressions, expressive language, rigidity). Participants also completed a timed version of the standard Raven’s Progressive Matrices test (Raven et al., 1996). Thirty participants were initially assigned to either high- or low-AT groups after performing a tertile split in the ranked SATQ scores of the initial pool of 47 participants. The final group characteristics after replacing one participant in the low AT group, and two participants in the high AT group (see below for details) can be seen in Table 1. As a comparison to the SATQ scores described in Table 1, SATQ scores in the general population have been reported with a mean of 23.1 (*SD* = 7.1), and in a clinical ASD sample with a mean of 40.8 (13.6; Kanne et al., 2012).

**Table 1:** Demographic information.

	*N*	Mean age (*SD*)	Mean Raven’s raw score (*SD*)	Gender ratio (M:F)	Mean SATQ score (*SD*)
Low autism trait group	15	24.7 (5.2)	48.4 (4.6)	4:11	10.6 (4.5)
High autism trait group	15	27.4 (9.4)	46.4 (5.8)	7:8	32.4 (5.5)

A power analysis to determine the sample size, utilizing the GPower package (Faul et al., 2009) was based on Crouzet et al. (2010), Bannerman et al. (2012) and Wilson et al. (2010). First for the within-subject comparison, when an effect size from Crouzet et al. (2010), dz = 2.5 was used, assuming a correlation among repeated measures (face and vehicle targets in saccadic choice reaction time tasks), *r* = 0.8, a sample size of four would be required for getting power of 0.80 with alpha level of 0.05. More conservatively however, when the effect size dz = 0.86 was estimated from Figure 3 of Bannerman et al. (2012) again assuming a correlation among repeated measures (upright and inverted fearful faces in a simple saccadic detection task), *r* = 0.8, a total sample size of 15 was calculated and aimed for in the current study. For the between-subject comparison, although not a saccadic reaction time task, Wilson et al. (2010) report a group effect for time to first fixation towards a face of approximately *d* = 1.16. Using the same assumptions as above, a sample size of 13 per group would be required.

### Stimuli and Procedure

Participants viewed stimuli on a PC 24-inch display monitor using Experiment Builder software, and saccades were recorded using the EyeLink 1000 Plus, a video-based eye-tracking system. Participants viewed tasks binocularly, positioned in a chinrest for stability 57 cm from the monitor.

Before commencing each task, a 9-point calibration of the eye movement recording system was carried out. After every 10 trials, a fixation drift-check was made to ensure that the difference between computed fixation position during calibration and the current target is not large.

Task design and procedure closely followed that of Crouzet et al. (2010). Photographs were all grayscale and consisted of 200 natural scenes containing a car, along with 200 natural scenes containing a face, and 200 distractor natural scenes containing neither a face nor a car. The car stimuli were sourced from the internet while the face stimuli and distractor natural scenes were provided by Crouzet et al. (2010). Each image was converted to grayscale, 330 × 330 pixels, and adjusted to a mean luminance value of 128 using Adobe Photoshop. Half the face stimuli were males and half were females. The faces and cars were positioned in different locations within the scene rather than predictably central and were also of varied size and viewpoint (i.e., front/side angle etc.). Most of the face and the car images consisted of close-up views of the target, with approximately 30% (face images) and 35% (car images) taken from mid-stance views. Each photo had a retinal size of 14° by 14° of visual angle and was always positioned 8° left or right of fixation.

**Figure 1:**
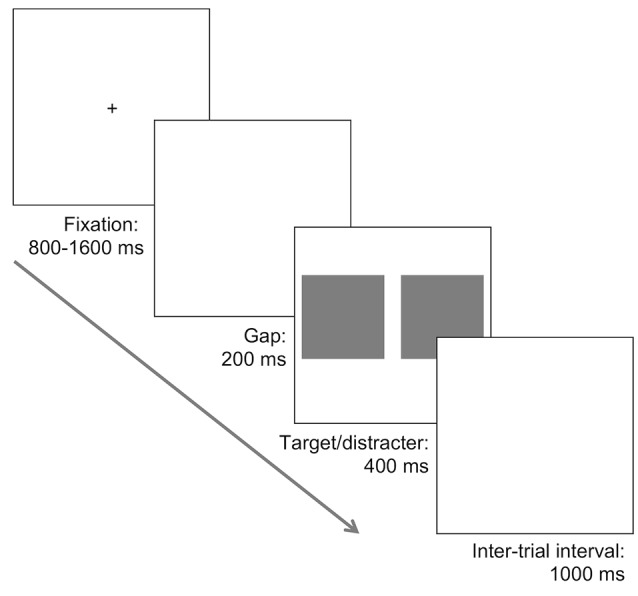
Illustration of the task procedure. A fixation was followed by a 200 ms blank screen, and subsequently two images were presented. In different tasks, one photograph contained a face or a car and was presented alongside a distractor photograph consisting of a natural scene with no car or face. In separate tasks both the target and distractor photographs were presented in an upright orientation, or inverted 180°. Participants were required to make a saccade towards the location of the target, presented randomly to the left or the right, as quickly and accurately as possible.

Each saccadic choice reaction-time task involved the presentation of two pictures, one on each side of fixation; one picture always contained the target (face or car), and the other was a natural scene that did not contain a target. The same set of 200 natural distractor scenes used for the face tasks were also used as the distractor images for the car tasks. The photo containing the target was randomly presented to the left or right. Initially, a black fixation cross on a white background was presented centrally for a random duration lasting between 800 and 1,600 ms, followed by a blank screen for 200 ms, and then the two images were presented for 400 ms followed again by a blank white screen (see Figure 1).

In the upright face task, participants were required to make a saccade towards the picture containing a face and completed 200 trials. Target and distractor images were both randomly sampled without replacement. This design was repeated in the inversion task, except that both target and distractor pictures were rotated 180°. Exactly the same procedure was used in the upright and inverted car target tasks. Thus, four separate tasks were completed, with task order counterbalanced between participants. Within each task, participants were allowed a brief break every 50 trials. The whole experiment lasted approximately 75 min.

### Data Analysis

Eye-movements were recorded monocularly at 1,000 Hz. Data processing and extraction made use of Eyelink Data Viewer software, before further processing in Excel, with final analyses utilizing SPSS. All saccades were recorded and defined by an eye-movement motion threshold of 0.2°, velocity above 30°/s, and acceleration greater than 8,000°/s^2^. An accurate response was defined as an eye movement of 4° or greater in the direction of the photo containing a target. For each participant, we calculated the percent correct trials on each task. Mean SOTs were analyzed for correct trials only, with saccades less than 4° or commencing earlier than 80 ms (i.e., indicating anticipation) excluded from the analysis. One participant in each AT group had missing data for a single task. Box plots for SOTs on each task and also the inversion effects for each target category revealed a high AT participant for face inversion, and a low AT participant for car inversion, both more than two standard deviations above the mean for their group. In order to maintain equal sample sizes, these participants were replaced with the participant next in the ranked SATQ data and formed the final sample for both accuracy and saccadic analyses (see Table 1). To analyze the accuracy and SOT data, separate three-way mixed ANOVA’s were conducted, including target (face, car) and orientation (upright, inverted) as within-subject factors, and group (low AT, high AT) as a between-group factor. Effect sizes are reported, adopting the convention of small, medium and large effects with partial eta squared values of 0.01, 0.06 and 0.14, respectively.

Given the possibility that apparent discrepancies in the gender balance between AT groups may have influenced the results, further analyses were conducted. Fisher’s exact test indicated that gender was not statistically imbalanced between AT groups (*p* = 0.45). Nevertheless, a mixed ANOVA using gender instead of AT group as the between-groups factor to analyze saccadic latencies did not reveal a main effect of gender (*p* = 0.219), nor any interactions including gender (*p*’s > 0.795). Together these analyses suggest that gender does not appear to have influenced saccadic latencies in the current study.

## Results

### Accuracy

Both groups performed the tasks with a high degree of accuracy (mean accuracy: low AT group—face upright = 98%, face inverted = 95%, car upright = 92%, car inverted 86%; high AT group—face upright = 96%, face inverted = 95%, car upright = 90%, car inverted 87%). A three-way mixed ANOVA revealed a main effect of target, *F*_(1,28)_ = 81.56, *p* < 0.001, $\eta_{\text{p}}^{2}$ = 0.744, with accuracy higher on face tasks than car tasks; a main effect of orientation, *F*_(1,28)_ = 26.75, *p* < 0.001, $\eta_{\text{p}}^{2}$ = 0.489, with accuracy higher for upright tasks than inverted tasks; but no effect of group, *F*_(1,28)_ = 0.08, *p* = 0.782, $\eta_{\text{p}}^{2}$ = 0.003. The only significant interaction was a two-way interaction between orientation and group, *F*_(1,28)_ = 5.43 *p* = 0.027, $\eta_{\text{p}}^{2}$ = 0.162, with simple effects analyses demonstrating that, although the low AT group was more accurate for upright than inverted stimuli (*p* < 0.001), the high AT group were not more accurate for upright than inverted stimuli (*p* = 0.054), regardless of target category.

### Saccadic Onset Times

**Figure 2:**
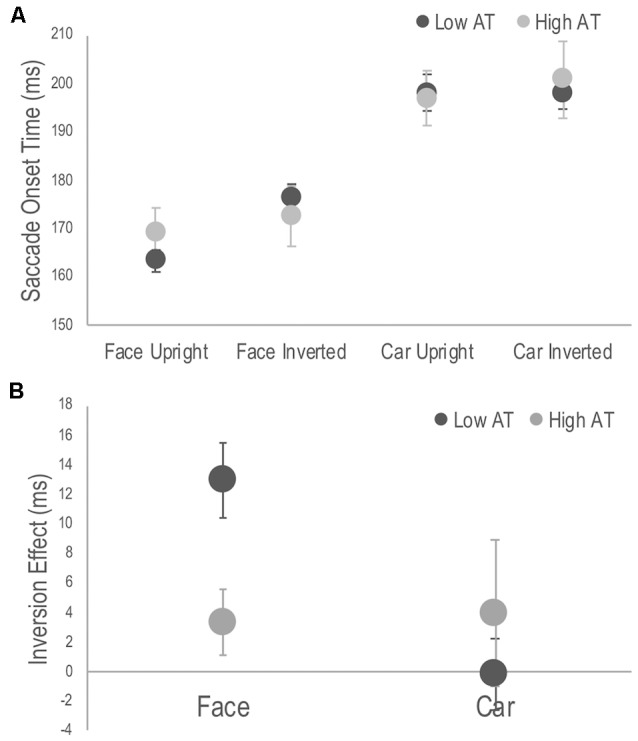
**(A)** Average saccade onset times (SOTs) to detect the photograph containing a face or a car in the upright and inverted tasks for high and low Autism Trait (AT) Groups. **(B)** Face and car inversion effects, calculated as the difference in mean SOTs between upright and inverted tasks for high and low AT Groups. Error bars indicate standard error of the mean. The low AT group demonstrated a significant face inversion effect (*p* < 0.001), whereas the high AT group did not (*p* = 0.170). The face inversion effect of the low AT group was significantly larger than that of the high AT group (*p* = 0.008). Neither group demonstrate a car inversion effect.

A three-way mixed ANOVA revealed significant main effects of target, *F*_(1,28)_ = 207.54, *p* < 0.001, $\eta_{\text{p}}^{2}$ = 0.881, with SOTs to faces faster than those to cars; orientation, *F*_(1,28)_ = 8.84, *p* = 0.006, $\eta_{\text{p}}^{2}$ = 0.24, with SOTs to upright targets faster than those to inverted targets; but not for group, *F*_(1,28)_ = 0.02, *p* = 0.878, $\eta_{\text{p}}^{2}$ = 0.001. There were no significant two-way interactions involving the group factor. The three-way interaction, however, was significant, *F*_(1,28)_ = 4.97, *p* = 0.034, $\eta_{\text{p}}^{2}$ = 0.151 (see Figure 2A). Simple main effects analysis used to interpret this interaction demonstrated that it was driven by differences in the inversion effects between groups. The inversion effect was defined as the paired comparison of SOTs between upright and inverted targets separately for each target category. Specifically, the low AT group showed a significant inversion effect for faces (*p* < 0.001) but not for cars (*p* = 0.968), whereas the high AT group did not demonstrate a significant face inversion effect (*p* = 0.170), nor a significant car inversion effect (*p* = 0.309; see Figure 2B). A *t*-test also revealed that the high AT group demonstrated a smaller face inversion effect compared with the low AT group, *t*_(28)_ = 2.85, *p* = 0.008. Although the high AT group showed a slower SOT for faces compared with the low AT group, the group difference was not significant (*p* = 0.301), and no other differences in SOTs were apparent between the groups for any of the tasks (*p*’s > 0.651; see Figure 2A).

Reaction time data, including saccadic reaction times, is usually positively skewed, and median SOT, rather than mean SOT per participant may be considered appropriate for the current analysis. Although this solution is not recommended for data sets in which conditions have different number of trials (Miller, 1988; as was the case here due to the exclusion of trials not meeting inclusion criteria), a mixed ANOVA was run as suggested by a reviewer. After replacing a further outlier in the high AT group, the 3-way interaction was still significant, *F*_(1,28)_ = 4.30, *p* = 0.047, $\eta_{\text{p}}^{2}$ = 0.133. Simple main effects demonstrated the same pattern of results as for the analysis based on means (see Appendix). Indeed the difference in SOT between the mean and median for each participant was small, ranging on average between 3–5 ms depending on the task condition.

## Discussion

The results of our experiment indicate that people with high (though sub-clinical) ATs showed different eye-movement patterns, compared with people with low ATs, when orienting towards faces in natural scenes. In particular, these differences were demonstrated by a reduced latency-based face inversion effect. These effects were found to be specific to the detection of faces with no evidence for differences in inversion effects when detecting inanimate targets, such as cars. Although the current study was not designed to test oculomotor and early-stage visual processing, the lack of a generalized impairment for saccade onset latencies and the specificity of the inversion effects to face targets suggests the oculomotor and visual processing deficits that have sometimes been reported in ASD populations across a range of paradigms are unlikely to account for the face-detection deficit we observed (for reviews, see Simmons et al., 2009; Freedman and Foxe, 2018).

Whereas low AT participants demonstrated a very pronounced face inversion effect, with faster SOTs towards upright than inverted faces, this effect was entirely absent in the participants with high ATs. Previous studies have found reduced or absent face inversion effects in ASD populations (McPartland et al., 2004; Rose et al., 2007) and in a neurotypical population a smaller face inversion effect was found to predict ATs (Wyer et al., 2012), although Weigelt et al. (2012) have cast doubt on the reliability of these findings. One difficulty in making comparisons between studies is that different measures of face processing have been used; for example, participants may be required to match face identity in some studies or expression in others. The reduced saccadic inversion effect reported here suggests that the timing of the activation of face processing mechanisms may be a critical variable, a conclusion that is also supported by the finding in an ASD sample of a reduced inversion effect in the N170 latency when viewing faces (McPartland et al., 2004).

Reports of a lack of face inversion effects in ASD (McPartland et al., 2004; Rose et al., 2007; Vettori et al., 2019) have been used to argue that individuals with ASD use a more feature-based approach to face processing. Typical observers are thought to use a more global or holistic approach to face processing, rather than a feature-by-feature analysis, and it is the disruption to this global analysis from turning the face upside down that produces the face inversion effect (Rossion, 2009). In the current study, a dramatic reduction in a saccade latency-based face inversion effect in participants with higher ATs suggests that this more feature-based approach to face processing extends across the broader autism spectrum. Importantly, it suggests that this feature-based approach operates not only at a slower more deliberate level but also at a faster and more reflexive level as required by the task demands in the current study. Finally, in both ASD and in the broader autism spectrum, previous studies have suggested either a more generalized impairment in global visual processing or a bias in local visual processing (Plaisted et al., 1999; Cribb et al., 2016). This might seem to be inconsistent with the current result given that different inversion effects were established for high and low AT groups when detecting faces, but not when detecting cars. Unlike for faces, however, it is assumed that the processing of non-face objects typically relies more on a feature-based strategy (Farah et al., 1998). As a consequence, reduced face inversion effects may reflect a face-specific anomaly in processing in the high AT participants, though a more general global processing deficit would be expected to be evident only when comparing the upright and inverted face conditions.

Although all participants were able to quickly and accurately select and direct eye-movements towards both face and car target categories, face targets were selected more accurately and with faster response times than for car targets. This replicates the findings in neurotypical adults by Crouzet et al. (2010), highlighting that faces constitute a special category of objects. Across all participants in the current sample, there was a 30-ms advantage in detecting a face as opposed to a car. It may be that humans have an innate specialized face specific module (Kanwisher and Yovel, 2006) or that a general-purpose object processing system develops expertise in face recognition given lifelong repeated exposure to faces (Hoehl and Peykarjou, 2012). A more interactive view suggests that innate tendencies to orient to faces lead to the development of face-sensitive regions in the ventral stream (de Haan et al., 2002). Regardless, it is evident that a clear behavioral advantage for the detection of faces is a strong and robust effect.

Although slower saccade initiation by high AT individuals to faces in a choice reaction time task was not established as expected, a moderate effect size ($\eta_{\text{p}}^{2}$ = 0.038) in the predicted direction was evident in this sub-clinical population. It remains possible that this effect would be more pronounced in a clinical sample and could reflect differences in conductance or processing speed within face regions of the ventral stream. Although a trend suggested that the high AT group was slower overall to respond to upright faces than was the low AT group, the saccadic onset in both groups was still faster than what may be expected from ERP studies that have shown an N170 potential evoked by faces in neurotypical adults (e.g., Eimer and Holmes, 2002). The rapid onset of face-directed saccades observed in all participants may be consistent, however, with other reports using ERP or magnetoencephalography (MEG) that have found face-selective activation occurring at 100–120 ms (Eimer and Holmes, 2002; Liu et al., 2002) or even as early as 30–60 ms after stimulus onset in the ventral stream (Braeutigam et al., 2001). It is interesting to note that early visual processing abnormalities have been reported in adults with ASD (compared to healthy control participants), with weaker and less lateralized MEG responses to faces occurring around 145 ms in the ventral stream of the right hemisphere as well as abnormal earlier responses at 30–60 ms over temporal sites (Bailey et al., 2005).

The fast activation of neural processes subserving rapid saccadic responses to faces that we and others have observed may also be explained by processing that passes through subcortical pathways, bypassing the visual cortex, through the superior colliculus, pulvinar, and amygdala (Johnson, 2005). A dynamic causal modeling study based on MEG data suggested that there might be a direct pulvinar-amygdala activation that occurs as early as 70 ms, regardless of emotion or spatial frequency filtering (McFadyen et al., 2017). Human intracranial recording has also established a similar rapid amygdala activation specific to the low spatial frequency components of fearful faces (Méndez-Bértolo et al., 2016). Crucially, ASD has been associated with structural and functional abnormalities in the amygdala (Baron-Cohen et al., 2000), indicating that rapid subcortical activation could explain differences in saccadic anomalies in the high AT group in the current study. Although the amygdala has typically been associated with threat detection, other work suggests that the amygdala mediates goal-directed relevance detection more generally (Sander et al., 2003). Thus, the temporal advantage that we observed in the detection of faces, even though they had a range of different expressions, could reflect the operation of these amygdala-based mechanisms.

Abnormalities in the magnocellular visual system have been reported in both ASD (e.g., Greenaway et al., 2013) and in neurotypical individuals with higher ATs (e.g., Crewther et al., 2015). The magnocellular system is the faster conducting of the two largely separate geniculostriate pathways and preferentially responds to stimuli with lower spatial- and higher temporal- frequencies (Laycock et al., 2007). The magnocellular system is also thought to drive the subcortical route through superior colliculus (Schiller et al., 1979), and, of particular interest here, has been linked to the fast subcortical face detection pathway that subserves automatic face processing (Johnson, 2005). A number of studies in neurotypical adults have demonstrated a link between subcortical activation for faces with low spatial frequencies expected to bias the magnocellular system (Johnson, 2005). There is also evidence for impairments in face and emotion processing in ASD being linked to the low spatial frequency content of face images (e.g., de Jong et al., 2008). Thus, although highly speculative with regard to the current data, it remains possible that anomalies in these face processing mechanisms *via* the superior colliculus, pulvinar amygdala pathway may contribute to the absence of a saccadic advantage for upright compared with inverted faces in the high AT participants.

One potential limitation of the current study is the issue of gender and the relatively lower number of males in the low AT group, despite this imbalance not reaching statistical significance. It should be noted that the direction of this imbalance is consistent with the higher incidence of males diagnosed with ASD (Fombonne, 2005) and the higher number of ATs endorsed by males than females in the general population (Baron-Cohen et al., 2001). Follow-up analyses did not show any effect of gender on task performance, a finding consistent with Wyer et al. (2012), though the current study was not sufficiently powered to include gender in the main analysis. Thus, although it does not appear to have done so, it remains possible that gender may have exerted some influence on the results.

In conclusion, the current study finds evidence for differences in saccadic processing between high and low AT participants, that appears to be specific to face detection, and is most pronounced in the examination of saccadic face inversion effects. This finding reinforces the suggestion that people with high ATs do not treat faces as a special category of object, and instead appear to process them in the same way as they do any object. Alternatively, it could be argued they may process any object as they do a face, though in either case, it appears both types of stimuli may have equal salience in these participants. Taken together then, our findings have revealed an anomaly in the automatic and rapid detection of upright compared with inverted faces in individuals with high ATs.

## Data Availability Statement

The datasets generated for this study are available on request to the corresponding author.

## Ethics Statement

The studies involving human participants were reviewed and approved by College of Science, Health & Engineering Human Ethics Sub-Committee, La Trobe University. The participants provided their written informed consent to participate in this study.

## Author Contributions

RL and MG developed the study concept. All authors contributed to the study design. Testing and data collection were performed by KW and AW. RL performed the data analysis. Data interpretation was made by RL, MG, and SC. KW and AW provided initial drafting of the manuscript, and RL completed the writing. MG and SC provided critical revisions. All authors approved the final version of the manuscript.

## Conflict of Interest

The authors declare that the research was conducted in the absence of any commercial or financial relationships that could be construed as a potential conflict of interest.

## Figures and Tables

**Figure A1:**
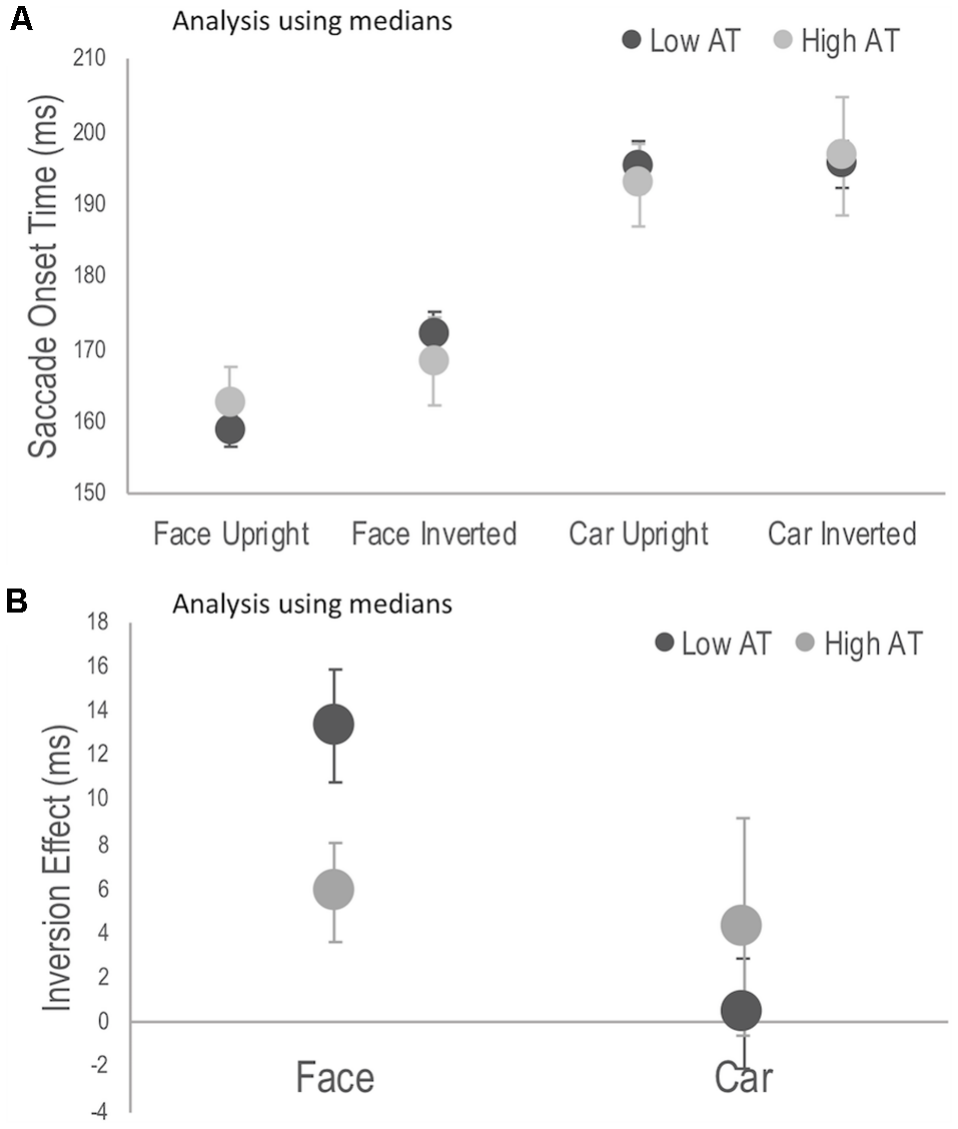
Reanalysis using individual median scores, rather than means as in Figure 2. **(A)** Median saccade onset times (SOTs) to detect the photograph containing a face or a car in the upright and inverted tasks for high and low Autism Trait (AT) Groups. **(B)** Face and car inversion effects, calculated as the difference in median SOTs between upright and inverted tasks for high and low Autism Trait (AT) Groups. Error bars indicate standard error of the mean. Error bars indicate SEM.
