# Corrigendum: Saccade Latency Provides Evidence for Reduced Face Inversion Effects With Higher Autism Traits

**DOI:** 10.3389/fnhum.2020.00058

**Published:** 2020-02-21

**Authors:** Robin Laycock, Kylie Wood, Andrea Wright, Sheila G. Crewther, Melvyn A. Goodale

**Affiliations:** ^1^School of Health and Biomedical Sciences, RMIT University, Melbourne, VIC, Australia; ^2^School of Psychology and Public Health, La Trobe University, Melbourne, VIC, Australia; ^3^The Brain and Mind Institute, The University of Western Ontario, London, ON, Canada

**Keywords:** autism, face processing, face inversion, saccade, eye-movements

In the original article, there was a mistake in the published legend for Figure 2. The results for the high and low Autism Trait (AT) groups were mistakenly interchanged. The correct legend appears below.

**Figure 2. (A)** Average saccade onset times (SOTs) to detect the photograph containing a face or a car in the upright and inverted tasks for high and low Autism Trait (AT) Groups. **(B)** Face and car inversion effects, calculated as the difference in mean SOTs between upright and inverted tasks for high and low AT Groups. Error bars indicate standard error of the mean. The low AT group demonstrated a significant face inversion effect (*p* < 0.001), whereas the high AT group did not (*p* = 0.170). The face inversion effect of the low AT group was significantly larger than that of the high AT group (*p* = 0.008). Neither group demonstrate a car inversion effect.

The authors apologize for this error and state that this does not change the scientific conclusions of the article in any way. The original article has been updated.

